# Patterns of Sedentary Behaviour in Female Office Workers

**DOI:** 10.3934/publichealth.2016.3.423

**Published:** 2016-06-24

**Authors:** Alison Kirk, Ann-Marie Gibson, Katie Laverty, David Muggeridge, Louise Kelly, Adrienne Hughes

**Affiliations:** 1 Physical Activity and Health group, School of Psychological Science and Health, Graham Hills Building, 40 George Street, University of Strathclyde, Glasgow, UK; 2 Exercise, Nutrition and Environment Research Group, School of Science and Sport, University of the West of Scotland, Hamilton, UK; 3 Department of Exercise Science, California Lutheran University, 60 W. Olsen Road, #3400, Thousand Oaks, CA 93065, USA

**Keywords:** sedentary behaviour, office workers, patterns, female, objective measurement

## Abstract

**Background:**

Prolonged sedentary behaviour is associated with poor health outcomes. Office workers often engage in excessive sedentary behaviour, however limited research reports on how this sedentary behaviour is accumulated. This study examines objectively measured patterns of prolonged sedentary behaviour in female office workers during weekdays and weekend days and across time of day.

**Methods:**

Full time female office workers from a Scottish University participated (N = 27 mean age 43.0 ± 11.5 yrs; BMI 25.8 ± 4.1 kg/m^2^). Participants wore an *activ*PAL™ for 7 days and completed a diary of waking and working hours. Average week and weekend time sitting, standing and stepping was calculated and also expressed as a proportion of waking day. Average week and weekend daily step count and sit to stand transitions were calculated. Continuous bouts of sedentary behaviour were categorised as: 20–40, 40–60 and > 60 minutes and compared between week and weekend days and across time of day.

**Results:**

Average weekday sitting time and proportion was higher (*P* < 0.05) than weekend days [9.1 hrs (66%) vs 8.1 hrs (56%)]. Time and proportion spent standing was higher (*P* < 0.01) at weekends than weekdays [4.6 hrs (32%) vs 3.8 hrs (27%)]. Time spent stepping [weekday 1.8 hrs (12%) vs weekend 1.7 hrs (12%)] and total daily step count (weekday 8903 vs weekend day 8014) were not significanlty different (*P* > 0.05) on weekdays vs weekend days. The pattern of sedentary behaviour bouts was different between week and weekend days. Week days were dominated by a consistent pattern of shorter (20–40 mins) sedentary behaviour bouts. The longest continuous sedentary behaviour bouts occurred in the evening, particularly at weekends.

**Conclusions:**

In office workers the most prolonged sedentary behaviour occurred in the evening, particularly at weekends. Interventions need to target these highly saturated periods of sedentary behaviour.

## 1. Introduction

Sedentary behaviour is characterised as waking activity in a sitting or reclining posture with a resultant energy expenditure of less than 1.5 metabolic equivalents [1]. Independent of physical activity levels, time spent in sedentary behaviours has been shown to be deleterious for health [2]. As technology is progressing, people are finding themselves in more social and occupational settings where time being spent sedentary is the dominant behaviour. Matthews, et al [3] reported adults spend approximately 70% of their waking day in sedentary behaviours and Kaci et al [4] reported that work time sitting accounted for more than half of the total daily sitting time on a work day (54%). Furthermore, research has identified “white-collar” workers [5],[6] and females [7] as being the most sedentary group of workers, indicating that female office based workers are a high risk group of people who engage in large amounts of sedentary time.

While there are clear and informative guidelines on physical activity, the guidelines for sedentary behaviour can be vague and often recommend that individuals should “minimise the amount of time spent being sedentary for extended periods” [8]. The lack of quantitative guidelines on the duration and intervals of sedentary behaviour leaves the general population to perhaps disregard the importance of how sedentary behaviour could negatively impact health and provides difficulty for people in judging how long an extended period of sedentary behaviour is.

Recent research has suggested in particular that long bouts of continuous sedentary behaviour negatively impacts health and breaking sedentary behaviour into smaller amounts of time, even if it accumulates to the same total duration, is more favourable for health [9]. Indeed, Healy, et al [9] has suggested that it is the pattern in which sedentary behaviour is accumulated that is the important aspect to study. Intervention studies, conducted in a controlled laboratory setting, where sedentary behaviour is interrupted every 20–30 minutes have reported improvements in metabolic biomarkers including waist circumference, glycaemic excursions, and triglycerides [10]. However to inform more specific guidelines on sedentary behaviour it is important to understand habitual patterns in sedentary behaviour and give consideration towards the feasibility and acceptability of implementing these intervention study findings. Advancement in methods of measuing sedentary behaviour with consideration of posture change and improved methods of device attachment to enhance participant compliance has increased the ability to accurately monitor patterns in sedentary behaviour over time [11].

The aim of this study was to explore objectively measured sedentary behaviour patterns in office workers during week and weekend days, and across time of day.

## 2. Materials and methods

### 2.1. Participants

27 participants volunteered and provided informed written consent to participate in the study (27 F; mean age 43.0 ± 11.5 years; BMI 25.8 kg/m^2^ ± 4.1 kg/m^2^). Participants were recruited from anywhere in the university and those who volunterred came from a variety of departments and sectors including academic (research and teaching) (n = 13) and administrative and professional services (n = 14). The participants were all female and full-time University office workers with no particular knowledge of sedentary behaviour. For all participants weekdays were work days and working hours occurred between 8 am and 6 pm. The study was approved by the University Ethics Committee at the University of Strathclyde and all procedures were conducted in accordance with the Declaration of Helsinki.

### 2.2. Procedures

On visit one participants completed informed consent and a brief demographic questionnaire and were fitted with an *activ*PAL monitor. Participants were also given a wear diary and were asked to record wake up and bedtime, working hours and any removal of the *activ*PAL and the reasons for removal (eg, irritation to the skin). After one week, participant returned the *activ*PAL and the wear diary.

### 2.3. Outcome measures

The *activ*PAL monitor has been well validated for measuring sedentary behavior with adults [12]. The monitor was worn 24 hours/day and standard procedures were followed to waterproof and attach the device (see http://www.paltech.plus.com/products.htm#activpal). The *activ*PAL monitor measures posture and classifies an individual's free-living activity into periods spent sitting or lying, standing and stepping. Sedentary time was calculated as monitor recorded sitting or lying time minus wear diary recorded sleep time. Wear diary sleep time was verified against monitor recorded activity. A minimum of four days of data were required to be included in analysis. If two or more of the same days (weekday or weekend day) were collected for the same participant, then the average of these days were used in analysis. Each participant included in the analysis for this paper had at least two weekdays and two weekend days.

### 2.4. Data analysis

Average total daily time spent in sedentary, standing and stepping activity, in addition to total daily step counts and sit to stand transitions were computed from the “summary by week” file generated from the *activ*PAL software for (1) all days combined, (2) weekday, (3) weekend day and (4) work time. This data is also reported as a proportion of the total waking day. Paired t tests were used to compare time spent in sedentary, standing and stepping activity, in addition to total steps and sit to stand transitions between weekday and weekend day data. The “events” file generated from the *activ*PAL software was then used to compute sedentary behaviour continuous bouts into the following durations: 20–40 minutes; 40–60 minutes and > 60 minutes. Bouts durations were chosen to capture short, medium and long sedentary bout events. This data was also expressed across time of day to explore when the longest continuous periods of sedentary behaviour occurred. All analyses were conducted using SPSS version 21. The number of bouts were normally distributed and differences are reported as statistically significant when *P* < 0.05.

## 3. Results

### 3.1. Participant characteristics

All participants provided valid *activ*PAL data of at least 4 days. Participants provided an average of 5.9 days of wear time, with an average of 4.4 week days and 2 weekend days of data.

### 3.2. Overall sedentary behaviour

The mean hours spent in sedentary, standing and stepping activity, in addition to total daily step counts and sit to stand transitions during all days combined, weekdays, weekend days and during working time is reported in Table 1.

**Table 1.** Sedentary behaviour during all days combined, weekdays, weekend days and during working time [mean (SD)].

Days	Wake/wear time (hrs)	Sedentary time (hrs)	Stand time (hrs)	Step time (hrs)	Step count	Sit to stand transitions
All days	14.6	8.7	4.1	1.8	9453	48
	(2.0)	(1.5)	(1.3)	(0.4)	(2589)	(15)
Week day	14.6	9.1	3.8	1.8	8903	48
	(2.1)	(1.6)	(1.2)	(0.5)	(2960)	(15.6)
Weekend day	14.4	8.1	4.6	1.7	8014	49
	(1.7)	(2.3)	(1.8)	(0.5)	(2758)	(18)
Working time	7.9	5.1	1.8	0.9	5045	24
	(0.9)	(1.1)	(0.7)	(0.5)	(2852)	(8.0)

Expressed as a proportion of the total waking day, for all days combined, participant were sedentary for 56%, standing for 32%, and stepping for 12% of the waking day. A paired t-test revealed that the average sedentary time on weekdays was higher (*P* < 0.05) than weekend days [9.1 hours (66%) versus 8.1 hours (56%)]. Time spent standing was higher (*P* < 0.01) at weekends than weekdays [4.6 hours (32%) versus 3.8 hours (27%)]. Time spent stepping [weekday 1.8 hrs (12%) vs weekend 1.7 hrs (12%)] and total daily step count (weekday 8903 vs weekend day 8014) were not significanlty different (*P* > 0.05) on weekdays vs weekend days. Participants spent 66% of their work time in sedentary activities, 23% standing and 11% stepping.

### 3.3. Bout pattern of sedentary behaviour

During a weekday, participants accumulated on average 5.1 sedentary bouts of 20 to 40 continuous minutes, 2.0 sedentary bouts of 40 to 60 continuous minutes and 1.8 sedentary bouts of at least 60 continuous minutes. During working time, participants accumulated on average 3.5 sedentary bouts of 20 to 40 continuous minutes, 1.4 sedentary bouts of 40 to 60 continuous minutes and 0.8 sedentary bouts of at least 60 continuous minutes. During weekend days participants accumulated on average 4.0 sedentary bouts of 20 to 40 continuous minutes, 1.6 sedentary bouts of 40 to 60 continuous minutes and 2.5 sedentary bouts of at least 60 continuous minutes. This pattern of continuous sedentary behaviour was different across weekdays and weekend days. Participants accumulated more 20 to 40 minutes bouts of sedentary behaviour during weekdays (*P* = 0.007) but accumulated more sedentary behaviour bouts of greater than 60 minutes during weekend days (*P* = 0.02).

**Figure 1.**
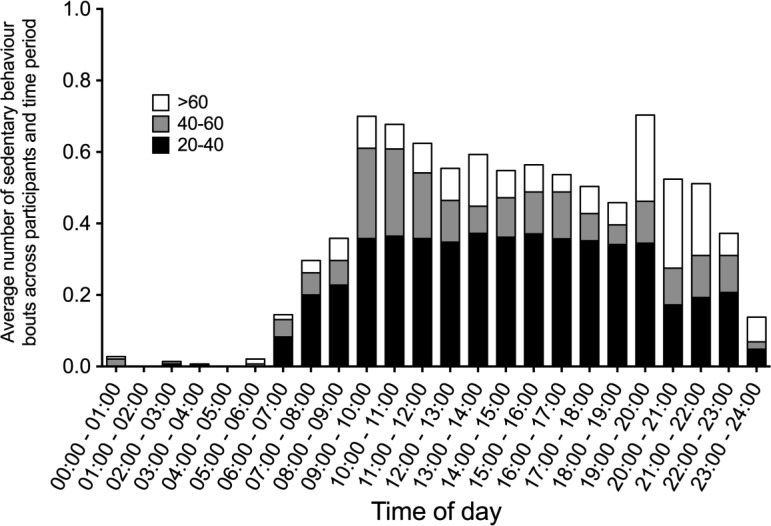
Pattern of continuous sedentary behaviour across time on weekdays.

**Figure 2.**
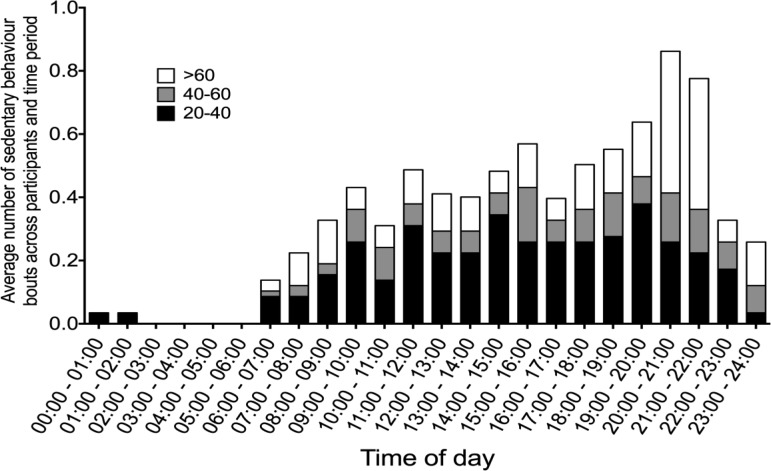
Pattern of continuous sedentary behaviour across time on weekends days.

Figure 1 and Figure 2 illustrate the pattern of continuous sedentary behaviour across time on weekdays and weekends days respectively. During weekdays, bouts of 20 to 40 minutes of sedentary behaviour are relatively constant between 9 am and 8 pm, with greater variation during weekends days. The most common period of day to be sedentary for 40–60 minutes was between 9 am and 11 am during weekdays, with more variation during weekend days (no distinct higher time periods). For sedentary behaviour bouts of greater than 60 minutes the most common time was between 7 pm and 10 pm during weekdays and between 8 pm to 10 pm during weekend days. The most common prolonged period of sedentary behaviour was bouts greater than 60 continuous minutes at weekend evenings between 8 pm and 10 pm.

## 4. Discussion

To date the majority of research exploring sedentary behaviour reports on overall sedentary behaviour, expressed as total hours per day or as a proportion of the overall or waking day. Continuous periods of sedentary behaviour are increasingly being recognised as a distinct health risk, however limited research reports on patterns of continuous sedentary behaviour [9]. Smith et al [13] analysed total amount of standing, sitting and stepping per hour over a 24 hour period which provides a summary of behaviour of each hour. However this does not provide information on the manner in which these behaviours are accumulated. Furthermore Chastin & Granat [14] have explored differences in pattern of sedentary behaviour across four subgroups of the population by utilising various parameters to express the distribution of sedentary behaviour bout within total sedentary time.

Overall the findings from this research suggest that during weekdays office workers sit more and stand less than during their weekend days. They accrue a similar step time, number of sit to stand transitions and step counts during week and weekend days. This summary of total sedentary behaviour is comparable to other published research [15],[16]. Clemes et al [15],[16] examined objectively measured sedentary behaviour and physical activity during and outside working hours in full time office workers. Sedentary time was higher on workdays. Furthermore individuals who were most sedentary at work were also more sedentary outside work time. This study advances current knowledge in providing a detailed analysis of patterns of sedentary behaviour during weekdays and weekend days and across time of day. Overall the pattern of continuous sedentary behaviour bouts appears to be different across weekdays and weekend days. Weekdays are dominated by a consistent pattern of shorter sedentary behaviour bouts (20–40 minutes). This is somewhat encouraging given the evidence suggesting that frequent interruptions in sedentary behaviour are beneficial to a variety of health markers [9]. Indeed, a recent laboratory based experimental study supports this and identified improvements in glucose metabolism even when sedentary behaviour was interrupted with brief, light activity [10]. A limitation in our study is not knowing the events which occurred during the interruptions of sedentary behaviour. Chastin et al [17] identified a number of important limitations within current research exploring the concept of breaks in sedentary behaviour and further research is required before consensus can be reached on what constitutes a “break” in sedentary behaviour and therefore how to categorise this in exploring patterns of sedentary behaviour.

The longest periods of continuous sedentary behaviour occur in the evening and in particular during weekend nights. This supports research conducted by Gardiner et al [18] and Smith, et al [13] who also found prolonged bouts of sitting more prevalent in later hours of the day. To date the majority of sedentary behaviour interventions have been focused within the workplace [19]. There is a need to complement this work with strategies and interventions targeting evening and weekend sedentary behaviour.

Study limitations include a small sample size of only females participants recruited from a university setting. Future research should aim to have larger samples and a balance of genders. The study however benefits from objective assessment of sedentary behaviour including postural assessment to classify standing as non-sedentary activity and detailed data processing to describe patterns of continuous sedentary behaviour. Furthermore monitors were waterproofed and worn continually for 7 days providing complete data to explore 24 hour movement patterns. Participant compliance with wearing the continuously wearing the *activ*PAL monitor was also very good.

## 5. Conclusions

Prolonged periods of sedentary behaviour are increasingly being recognised as a distinct health risk. This study helps identify where the most saturated periods of sitting occur during the day and therefore provides important information about when to target interventions.
